# Causal associations between ankylosing spondylitis and cardiovascular disease: a mendelian randomization analysis

**DOI:** 10.1016/j.clinsp.2026.100918

**Published:** 2026-04-02

**Authors:** Lei Zhou, Yong Tang, Jihong Jiang

**Affiliations:** Department of Orthopedics, The Affiliated People’s Hospital of Ningbo University, Ningbo City, Zhejiang Province, China

**Keywords:** Ankylosing spondylitis, Cardiovascular disease, Mendelian Randomization, Bioinformatics

## Abstract

**Background:**

Accumulating evidence suggests an association between Ankylosing Spondylitis (AS) and Cardiovascular Disease (CVD), yet the causal effects of AS on different CVD types remain unclear. This study aimed to determine the associations between AS and CVD using two-sample Mendelian Randomization (MR) analysis.

**Methods:**

The genetically predicted causal relationships between AS and six common cardiovascular and cerebrovascular diseases (hypertension, atrial fibrillation, acute myocardial infarction, coronary atherosclerosis, angina, and heart failure) were evaluated using MR analysis. Data originated from Genome-Wide Association Studies (GWAS). Single Nucleotide Polymorphisms (SNPs; *p* < 5 × 10^–8^, *r*^2^ < 0.01) were selected as Instrumental Variables (IVs) for MR analysis. The primary outcome for causal inference was the Inverse Variance-Weighted (IVW) method, complemented by other methods (MR Egger, weighted median, simple mode, weighted mode). All results were presented as Odds Ratios (ORs). Heterogeneity and pleiotropy analyses were performed to ensure result stability and reliability.

**Results:**

MR results provided positive causal evidence for AS on hypertension (OR = 1.0143; 95% CI: 1.0038‒1.0248, *p* = 7.269 × 10^–3^) and heart failure (OR = 1.0128; 95% CI: 1.0037‒1.0217, *p* = 5.76 × 10^–3^). Conversely, none of the IVW methods indicated causal effects for AS on the other four diseases. Sensitivity analyses supported the robustness of the results.

**Conclusion:**

This study indicates that ankylosing spondylitis has positive causal effects on hypertension and heart failure.

## Highlights

• MR analysis shows AS causally linked to hypertension & heart failure.
• No causal effects of AS on atrial fibrillation, AMI, CA, or angina.
• Inflammation may mediate AS-CVD relationship.

## Introduction

Ankylosing Spondylitis (AS) is a chronic inflammatory disease belonging to the Spondyloarthritis (SpA) family, characterized by inflammation in ligaments, peripheral joints, tendons in the spine, and other extra-articular organs such as the eyes, skin, and cardiovascular system. AS typically initiates in the sacroiliac joint, progressing to spine fusion (known as “bamboo spine”), leading to limited mobility, pain, and spinal deformity.^1^ AS onset commonly occurs during the second or third decade of life, with a male-to-female ratio of 3:1.^2^ The prevalence of AS ranges from 0.1% to 1.4%, varying across ethnic groups, with higher rates in non-mixed Caucasian populations.^3^ Despite extensive research, the exact pathogenesis of AS remains elusive due to its complexity. AS is considered a familial hereditary disease, with over 90% determined by genetic factors.^4^ Human Leucocyte Antigen-B27 (HLA-B27) is positive in ∼95% of AS patients, yet other non-HLA genes account for over 50% of the total AS risk, including IL-6R, IL-10, CXCR2, and NOTCH1.^5^ These genes, alongside AS-induced inflammation, may impact the cardiovascular system, albeit indistinctly. Infection and inflammation are the most frequent causes of death in AS patients.^6^

Cardiovascular Disease (CVD) is the leading cause of mortality globally.^7^ In 2019, an estimated 113 million people in European countries lived with CVD, with 12.7 million new cases reported annually.^8^ A recent study revealed that the lifetime risk of developing CVD is similar for men and women, with approximately two out of three individuals aged around 55 developing CVD during their remaining lifespan.^9^ A six-year follow-up report in the UK showed that the initial presentation of CVD was often heart failure, angina pectoris, transient ischemic attack, peripheral arterial diseases, or coronary atherosclerosis, rather than myocardial infarction or ischemic stroke.^10^ The prevalence of CVD has increased worldwide over the past decades, posing significant challenges.

Mendelian Randomization (MR) is a reliable approach for estimating causality between exposures and outcomes using genetic variants as IVs. MR studies are less susceptible to potential confounders and reverse causation because genotypes precede disease onset and are unrelated to environmental or lifestyle factors.^11^ Thus, MR has been widely used in causal inference studies. Several studies have shown that AS patients have a higher risk of developing CVDs.^12^, ^13^, ^14^, ^15^ However, a UK-based study concluded that vascular morbidity was not significantly increased in AS based on population data.^16^ In this study, we used SNPs as IVs to perform an MR analysis to explore the potential causal associations between AS and CVD risk.

## Materials and methods

### Study design

**Fig. 1:**
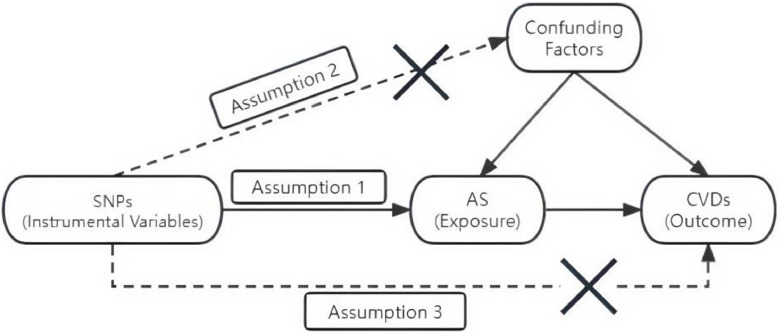
The MR schematic diagram.

To assess the causal associations between AS and CVDs, we conducted a two-sample MR analysis adhering to three key assumptions: (i) The SNPs considered as IVs are closely related to AS; (ii) No associations exist between SNPs and confounders of AS and CVDs; (iii) SNPs affect CVDs solely through the AS pathway. This two-sample MR analysis explored causal associations between AS and six common CVDs: Heart Failure (HF), hypertension, Atrial Fibrillation (AF), Coronary Atherosclerosis (CA), acute Myocardial Infarction (AMI), and angina (Fig. 1).

### Data source

SNP data for AS and the six CVDs were obtained from published GWAS, which had obtained relevant ethical approval. Thus, additional ethical approval or consent was not required. Detailed information on the data used is shown in Table 1 (available at https://gwas.mrcieu.ac.uk/ (accessed on 6 January 2023)). Because of the large sample sizes were prioritized to enhance statistical power for detecting genetic associations with AS and CVDs. This aligns with GWAS best practices, where larger samples improve reliability of effect size estimates and reduce false positives. And European ancestry cohorts minimize stratification bias, ensuring consistent genetic interpretation. Moreover, Public datasets have rigorous QC and ethical review, ensuring reproducibility.

**Table 1:** Descriptions for data sources used in analysis.

Traits	Data sources	Case	Control	Population	Access link
Exposure					
AS	FinnGen	1462	164,682	European	https://gwas.mrcieu.ac.uk/datasets/finn-b-M13_ANKYLOSPON/
Outcome					
HP	FinnGen	55,917	162,837	European	https://gwas.mrcieu.ac.uk/datasets/finn-b-I9_HYPTENS/
HF	GWAS Catalog	47,309	930,014	European	https://gwas.mrcieu.ac.uk/datasets/ebi-a-GCST009541/
AF	FinnGen	22,068	116,926	European	https://gwas.mrcieu.ac.uk/datasets/finn-b-I9_AF/
AMI	United Kingdom Biobank	2321	460,689	European	https://gwas.mrcieu.ac.uk/datasets/ukb-b-3469/
CA	FinnGen	23,363	195,429	European	https://gwas.mrcieu.ac.uk/datasets/finn-b-I9_CORATHER_EXNONE/
Angina	FinnGen	18,168	187,840	European	https://gwas.mrcieu.ac.uk/datasets/finn-b-I9_ANGINA/

AS, Ankylosing Spondylitis; HP, Hypertension; HF, Heart Failure; AF, Atrial Fibrillation; AMI, Acute Myocardial Infarction; CA, Coronary Atherosclerosis.

### Instrumental variables selection

SNP data for AS were derived from a published GWAS analyzing 166,144 European individuals. Genome-wide significant (*p* < 5 × 10^–8^) SNPs were extracted as IVs. Linkage Disequilibrium (LD) testing ensured SNP independence (*r*^2^ < 0.01)). SNPs associated with confounding factors or exhibiting LD were removed. F-statistics were calculated, and weak IVs (*F* < 10) were excluded.

### Statistical analysis

Five MR analysis methods assessed potential associations between AS and CVDs: IVW, MR Egger, weighted median, simple mode, and weighted mode. IVW was the primary method due to its consistency in estimating causal effects even with heterogeneity.^17^^,^^18^ The other methods complemented IVW. MR Egger assessed horizontal pleiotropy and causal effect reliability.^19^ Simple mode and weighted mode further assessed associations, while the weighted median provided reliable causal estimation if > 50% of IVs were valid. Cochran’s Q test and MR-Egger intercept tested heterogeneity and pleiotropy.^20^ When MR-Egger intercept p-values > 0.05, pleiotropic effects were considered absent. Leave-one-out sensitivity tests evaluated MR result reliability and effectiveness (statistical significance at *p* < 0.05). All analyses were performed using the Two-Sample MR package in R version 4.4.2.

## Results

**Fig. 2:**
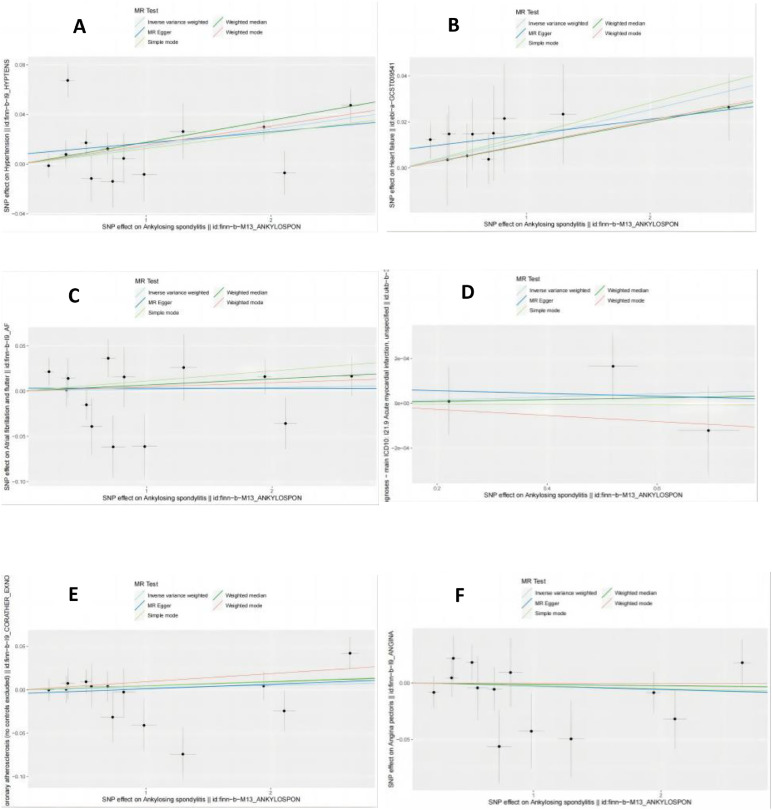
The MR scatter plot about the causal effect of AS on 6 different cardiovascular diseases. (A) Hypertension; (B) Heart failure; (C) Atrial fibrillation; (D) Acute myocardial infarction;(E) Coronary atherosclerosis; (F) Angina.

**Fig. 3:**
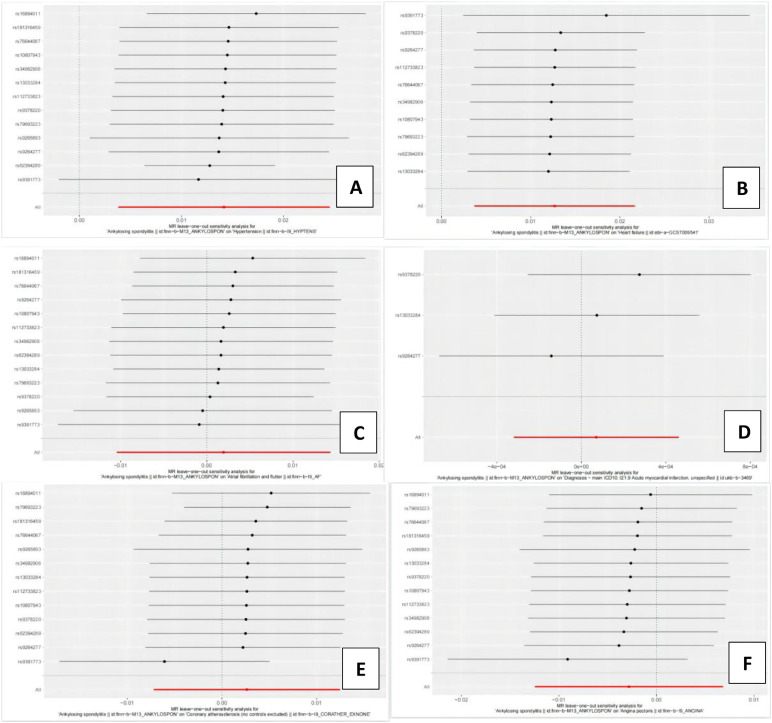
Leave-one-out analysis for the causal effect of AS on 6 different cardiovascular diseases. (A) Hypertension; (B) Heart failure; (C) Atrial fibrillation; (D) Acute myocardial infarction;(E) Coronary atherosclerosis; (F) Angina.

Detailed MR results for AS on the six CVDs are listed in Table 2. The F-statistic of SNPs calculated in Table 2 were all over 10. The F-statistics for each SNP are provided in Table 2. The threshold of *F* > 10 was used as it is conventional in MR studies, although we acknowledge that F-statistics marginally above 10 may still indicate weak instruments. Sensitivity analyses were conducted to confirm instrument strength, and these results are reported in Table 3. IVW results indicated positive causal effects of AS on hypertension (OR = 1.014; 95% CI: 0.0038‒0.0245; *p* = 7.269 × 10^–3^) and heart failure (OR = 1.013; 95% CI: 0.0037‒0.0217; *p* = 5.76 × 10^–3^). Complementary methods supported these findings. No heterogeneity, pleiotropy, or instability was observed (Fig. 2, Fig. 3, Table 3). No significant associations were found between AS and the other five CVDs. Pleiotropy was assessed using the MR-Egger intercept test. SNPs with a MR-Egger intercept p-value < 0.05 were considered pleiotropic and were excluded from the analysis.

**Table 2:** The Mendelian randomization analysis results with regard to causal effect of AS on 6 cardiovascular diseases.

Outcome	Methods	SNP(n)	OR	OR 95% CI	p-value
Hypertension	MR Egger	13	1.0091	0.9929‒1.0255	0.2953
	Weighted median	13	1.0179	1.0097‒1.0261	1.672 × 10^–5^
	Inverse variance weighted	13	1.0143	1.0038‒1.0248	7.269 × 10^–3^
	Simple mode	13	1.0126	0.9966‒1.0289	0.1498
	Weighted mode	13	1.0155	1.0073‒1.0237	2.864 × 10^–3^
Heart Failure	MR Egger	10	1.0066	0.9937‒1.0197	0.3456
	Weighted median	10	1.0101	1.0001‒1.0202	0.0485
	Inverse variance weighted	10	1.0128	1.0037‒1.0219	0.0058
	Simple mode	10	1.0143	0.9934‒1.0356	0.2149
	Weighted mode	10	1.0104	0.9993‒1.0216	0.0988
Atrial Fibrillation	MR Egger	13	0.9999	0.9802‒1.0199	0.9910
	Weighted median	13	1.0067	0.9936‒1.0200	0.3164
	Inverse variance weighted	13	1.0019	0.9896‒1.0144	0.7589
	Simple mode	13	1.0111	0.9849‒1.0381	0.4252
	Weighted mode	13	1.0046	0.9927‒1.0167	0.4635
Acute myocardial infarction	MR Egger	3	0.9999	0.9988‒1.0011	0.9321
	Weighted median	3	1.0000	0.9996‒1.0005	0.8596
	Inverse variance weighted	3	1.0001	0.9997‒1.0005	0.7230
	Simple mode	3	1.0000	0.9993‒1.0007	0.9799
	Weighted mode	3	0.9999	0.9992‒1.0005	0.7115
Coronary atherosclerosis	MR Egger	13	1.0053	0.9897‒1.0212	0.5219
	Weighted median	13	1.0046	0.9920‒1.0174	0.4752
	Inverse variance weighted	13	1.0026	0.9928‒1.0125	0.6001
	Simple mode	13	1.0049	0.9820‒1.0284	0.6854
	Weighted mode	13	1.0094	0.9985‒1.0203	0.1163
Angina	MR Egger	13	0.9970	0.9816‒1.0126	0.7092
	Weighted median	13	0.9988	0.9862‒1.0116	0.8554
	Inverse variance weighted	13	0.9972	0.9876‒1.0068	0.5653
	Simple mode	13	0.9976	0.9757‒1.0200	0.8333
	Weighted mode	13	0.9998	0.9878‒1.0121	0.9805

**Table 3:** The results of heterogeneity and pleiotropy test.

Variable	Heterogeneity test						Pleiotropy test	
	MR Egger			Inverse variance weighted			MR Egger	
	Q	Q_df	Q_pval	Q	Q_df	Q_pval	Intercept	p-val
HP	29.94	11	0.0016	31.74	12	0.0015	7.97×10^–3^	0.4329
HF	1.49	8	0.9928	3.15	9	0.9579	8.00×10^–3^	0.2338
AF	17.14	11	0.1038	17.25	12	0.1405	3.21×10^–3^	0.7948
AMI	1.33	1	0.2482	1.41	2	0.4929	6.91×10^–5^	0.8456
CA	15.30	11	0.1694	15.56	12	0.2122	−4.16×10^–3^	0.6702
Angina	12.13	11	0.3540	12.13	12	0.4353	3.16×10^–4^	0.9735

## Discussion

This study aimed to explore the relationships between AS and six CVDs using two-sample MR analysis. It may be the first to assess causal effects of AS on CVDs using GWAS datasets. All data in this study were derived from European GWAS databases. It is important to acknowledge that the generalizability of these findings to non-European populations may be limited due to differences in AS prevalence and genetic architecture across different ethnic groups. Future studies should aim to replicate these findings in diverse populations.

In the presence of pleiotropy, as suggested by the MR-Egger intercept test, we prioritized the MR-Egger and weighted median methods over the IVW method. This approach was taken to ensure more robust causal inference, particularly when the assumption of no pleiotropy may be violated. Heterogeneity was addressed using random-effects IVW. This method accounts for potential heterogeneity between SNPs and provides more conservative estimates of causal effects. The results from random-effects IVW are reported in Table 2.

Our results provide strong genetic evidence that AS has positive causal effects on hypertension and heart failure but not on atrial fibrillation, Acute Myocardial Infarction (AMI), coronary atherosclerosis, or angina pectoris. Although the ORs for hypertension and heart failure were statistically significant, their clinical significance is minimal. This requires interpretation in genetic contexts. Public health impact emerges through population-level risk accumulation in genetically predisposed subgroups (HLA-B27 carriers), where small per-unit effects amplify over time. Clinically, integrating genetic liability scores with traditional risk factors enables precise risk stratification in ankylosing spondylitis patients. For example, combining the 1.4% absolute risk increase with hypertension duration improves prediction accuracy. This supports targeted surveillance (annual cardiovascular screenings in high-genetic-risk groups) and aligns with precision medicine by refining intervention thresholds through gene-environment interaction analysis. The 95% CIs suggest a negligible absolute risk increase. For example, the OR of 1.014 for hypertension implies a very small increase in risk per unit increase in the genetic liability score. The small sample size for AMI (*n* = 2321 cases) is a significant limitation. This constrained statistical power may increase the risk of Type II errors, particularly in detecting subtle associations between AS and AMI. While our analysis found no significant causal relationship, this result should be interpreted given the potential for undetected true effects due to limited sample size. To address this limitation, a post-hoc power analysis may be conducted in the future. This would quantify our study's ability to detect clinically relevant effect sizes for AMI and angina outcomes, providing critical context for interpreting negative results. It would help distinguish between truly absent associations versus insufficient statistical power, thereby strengthening the validity of our conclusions regarding cardiovascular outcomes in AS patients.

Cardiovascular diseases have caused plenty of problems. In recent decades, the incidence of hypertensive, rheumatic and other heart disease is apparently on the rise in Europe.^21^ With the development of medicine, we also have further researches on ankylosing spondylitis as an inflammatory disease. The observed causal relationships between AS and hypertension/heart failure may be mediated through inflammation-driven pathways. AS is characterized by chronic inflammation, and elevated levels of pro-inflammatory cytokines, such as IL-6, TNF-α, and IL-17, have been implicated in both AS and cardiovascular pathology. These cytokines can contribute to endothelial dysfunction, vascular remodeling, and atherosclerosis, thereby increasing the risk of hypertension and heart failure. IL-6 activates signaling in vascular smooth muscle cells, promoting proliferation and vascular remodeling that directly elevates blood pressure. Elevated TNF-α in heart failure correlates with mitochondrial dysfunction and apoptotic cardiomyocyte loss. Recent basic and clinical researches indicated potential relationships exiting between the ankylosing spondylitis and cardiovascular diseases. A research using 36-month look-back window to collect data on baseline comorbidities, including hypertension, Peripheral Vascular Disease (PVD), Coronary Artery Disease (CAD) and other diseases, for people in both the AS and comparison groups showed that the risk for vascular mortality was increased >6-fold in patients with AS.^22^ A cross-sectional study proposed CV risk factors in patients with AS may correlate not only with CV disease, but also with disease activity.^23^ The ASAS-CoMoSpA study also indicated higher prevalence of hypertension, diabetes, ischemic heart disease, dyslipidemia, obesity in AS patients.^24^ As known, AS is a chronic inflammatory arthritis which can release inflammatory factors affecting the organs and spines. Therefore, the contention that inflammation underlies CV risk factors is also supported by some research.^25^ However, whether there is an exact relationship between the ankylosing spondylitis and cardiovascular diseases is unclear. Furthermore, these studies could only figure out the correlations but not causal relationships between AS and CVDs.

In our work we conducted various analyses to assess the OR 95% CI and p-value of AS on 6 common cardiovascular diseases based on a large sample size in GWAS. The genetic variants were taken as the IVs to imitate the RCT design, which were robust associated SNPs selected from large sample size. The results revealed causal effects of AS on hypertension and heart failure, while no causal associations between AS on atrial fibrillation, acute myocardial infarction, coronary atherosclerosis and angina pectoris. These results provided genetic evidence about the causal relationships between AS and different CVDs. Also, the two-sample MR we used in this study is a genetic epidemiology method, which can conquer various unmeasured confounding and make more accurate calculations. Therefore, we consider these causal inferences are reliable and robust. Additionally, some limitations were existed in our study. First, the use of European GWAS data limits the generalizability of our findings to other ethnic groups. Second, the smaller sample size for AMI may have reduced our power to detect causal effects. Third, while we have attempted to address pleiotropy, residual confounding cannot be entirely ruled out. Finally, the clinical significance of our findings, particularly for hypertension and heart failure, is minimal despite statistical significance.

## Conclusions

This Mendelian randomization analysis provides positive evidence that patients with ankylosing spondylitis may increase the occurrence of hypertension and heart failure but have no causal relationships with the other four CVDs studied.

## Authors’ contributions

Lei Zhou and Yong Tang wrote the first draft of the manuscript, reviewed and edited the manuscript and approved the final version of the manuscript. The other authors operated the formal analysis and data curation.

All authors have read and agreed to the published version of the manuscript.

## Data availability

The datasets generated and/or analyzed during the current study are available from the corresponding author upon reasonable request.

## Funding

This work is supported by the Ningbo Health Science and Technology Plan Project (2024Y49); Yinzhou District Health Science and Technology Plan Project (2025Y02).

## Declaration of competing interest

The authors declare no conflicts of interest.
